# The Beat

**DOI:** 10.1289/ehp.120-a60b

**Published:** 2012-02-01

**Authors:** Erin E. Dooley

## The “Flume Room”

The University of Michigan has created a specialized laboratory containing 150 artificial mini-streams, or “flumes,” that mimic a variety of river conditions as closely as possible.^^1^^ The flumes are populated with rocks, sediment, biota, and more than 3,000 gallons of water from the Huron River. The goal is to better understand how different stressors—nutrient and chemical pollution, exotic species, species extinctions, climate change, and erosion, for instance—affect river health and “ecosystem services” provided by the Huron such as pollutant decomposition and oxygen production.

**Figure:**
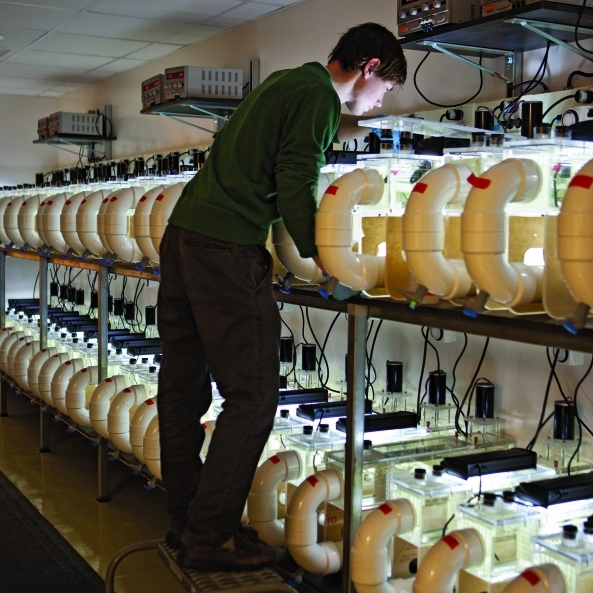
A University of Michigan researcher in the “Flume Room.” © 2011 University of Michigan Photo Services

## New Data on Beijing Air Pollution to Be Released

Beijing’s Municipal Environmental Protection Bureau has announced it will commence publishing hourly readings for PM_2.5_, PM_10_, sulfur dioxide, and nitrogen dioxide on its website.^^2^^ PM_2.5_ data—considered a better gauge of air quality than the PM_10_ data used to this point by the Chinese government—used to be available only for laboratory use and were not disclosed to the public. Beijing currently has 6 PM_2.5_ monitoring stations; additional stations will be instated before the end of 2012.

## What Happened to Pollutants after the BP Deepwater Horizon Spill?

Investigators have modeled how the underwater topography, currents, and bacterial populations in the Gulf of Mexico helped clear away constituents of the oil spilled during the BP *Deepwater Horizon* disaster in 2010.^^3^^ The researchers determined that the geography of the Gulf was key to keeping 50-mile-long eddies of microbe-laden water swirling over the site of the spill, continually introducing fresh loads of hydrocarbons to bacterial communities primed to degrade them by the initial load.

**Figure:**
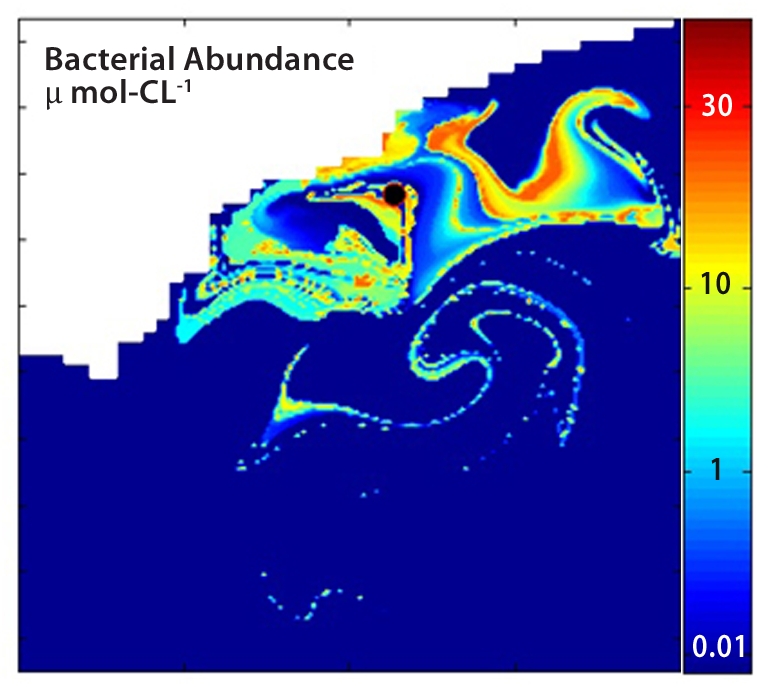
A new model unifies divergent theories about the fate of the oil spilled by BP *Deepwater Horizon* PNAS/ David Valentine and Igor Mezic

## Fukushima Cleanup Could Have Huge Environmental Impact

A year after Japan’s Tohoku earthquake, tsunami, and nuclear meltdown, Japanese officials have begun a massive radioactive contamination cleanup that will require clearing at least 1,000 km^2^ of land at an estimated cost of more than US$12.8 billion dollars.^^4^^ However, removing the estimated 15–31 m^3^ of contaminated soil and debris could destroy ecosystems and make areas vulnerable to flooding. The cleanup also raises the issue of where the large amounts of radioactive waste will be stored. Work is being carried out at 19 model sites to help determine the most efficient and effective methods for large-scale decontamination.

## New European Rules on Phosphorus in Detergents

In December 2011 the European Parliament approved new rules on phosphorus that will limit the amount to 0.5 g per single use of laundry detergent and 0.3 g per single use of dish detergent.^^5^^ The rules will take effect in June 2013 and January 2017, respectively. Many U.S. states began regulating phosphorus in laundry detergent in the 1980s. Dish detergent has been harder to reformulate, but in 2010 the American Cleaning Institute^®^ adopted voluntary limits on phosphorus in this product as well.^^6^^ Phosphorus discharged from households can contribute to harmful algal blooms in water bodies.

**Figure:**
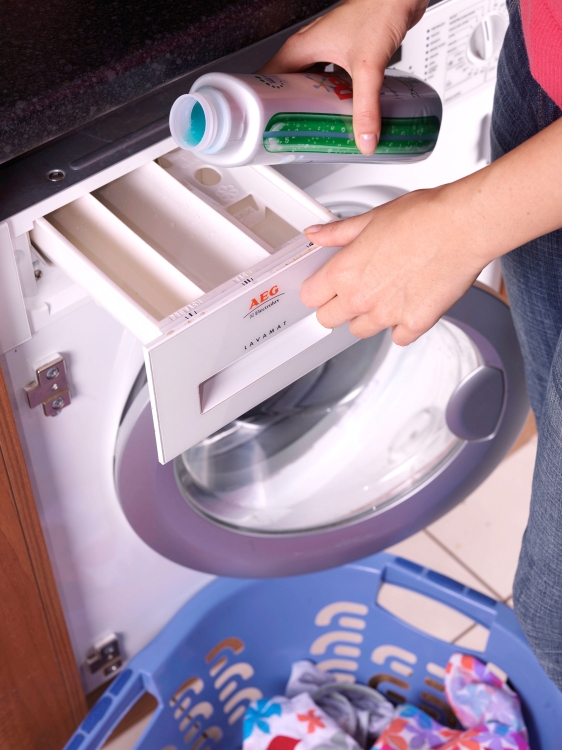
European householders will use low-phosphorus detergents in the near future. © mediablitzimages (UK) Limited/Alamy
